# Early-Life Exposure to Famine and Risk of Metabolic Associated Fatty Liver Disease in Chinese Adults

**DOI:** 10.3390/nu13114063

**Published:** 2021-11-13

**Authors:** Jing Liu, Guimin Wang, Yiling Wu, Ying Guan, Zhen Luo, Genming Zhao, Yonggen Jiang

**Affiliations:** 1 Disease Control Division, Songjiang District Center for Disease Control and Prevention, Shanghai 201600, China; jing_lj26@163.com (J.L.); songjiangwgm@163.com (G.W.); aries2119@163.com (Y.W.); amy30@163.com (Y.G.); emily040722@163.com (Z.L.); 2 The Key Laboratory of Public Health Safety of Ministry of Education, Department of Epidemiology, School of Public Health, Fudan University, Shanghai 200032, China; gmzhao@shmu.edu.cn

**Keywords:** early-life exposure, famine, metabolic associated fatty liver disease, sex-specific association

## Abstract

Background: Early-life exposure to the Chinese famine has been related to the risk of obesity, type 2 diabetes, and nonalcoholic fatty liver disease later in life. Nevertheless, the long-term impact of famine exposure on metabolic associated fatty liver disease (MAFLD), a recently proposed term to describe liver disease associated with known metabolic dysfunction, remains unknown. The aim of our study was to explore the relationship between early famine exposure and MAFLD in adulthood. Methods: A total of 26,821 participants (10,994 men, 15,827 women) were recruited from a cohort study of Chinese adults in Shanghai. We categorized participants into four famine exposure subgroups based on the birth year as nonexposed (1963–1967), fetal-exposed (1959–1962), childhood-exposed (1949–1958), and adolescence-exposed (1941–1948). MAFLD was defined as liver steatosis detected by ultrasound plus one of the following three criteria: overweight/obesity, type 2 diabetes, or evidence of metabolic dysregulation. Multivariable logistic regression models were performed to examine the association between famine exposure and MAFLD. Results: The mean ± standard deviation age of the participants was 60.8 ± 6.8 years. The age-adjusted prevalence of MAFLD was 38.3, 40.8, 40.1, and 36.5% for the nonexposed, fetal-exposed, childhood-exposed, and adolescence-exposed subgroups, respectively. Compared with nonexposed participants, fetal-exposed participants showed an increased risk of adulthood MAFLD (OR = 1.10, 95% CI 1.00–1.21). The significant association between fetal famine exposure and MAFLD was observed in women (OR = 1.22, 95% CI 1.08–1.37), but not in men (OR = 0.88, 95% CI 0.75–1.03). In age-balanced analyses combining pre-famine and post-famine births as the reference, women exposed to famine in the fetal stage still had an increased risk of MAFLD (OR = 1.15, 95% CI 1.05–1.26). Conclusions: Prenatal exposure to famine showed a sex-specific association with the risk of MAFLD in adulthood.

## 1. Introduction

Metabolic associated fatty liver disease (MAFLD), formerly named nonalcoholic fatty liver disease (NAFLD), is the most prevalent chronic liver disease worldwide. This disease affects nearly a quarter of the global population, posing major health and economic burdens to many societies, with no approved pharmacotherapy thus far [1]. The term MAFLD was proposed by an international panel of experts in 2020 because of a pressing need to update the nomenclature to more accurately describe liver disease associated with known metabolic dysfunction, particularly with the high prevalence of the disease and poor metabolic health, even among non-obese individuals [2,3,4]. Based on a better understanding of the pathogenic process underlying MAFLD, a set of “positive” diagnostic criteria for MAFLD was established regardless of alcohol consumption or other concomitant liver diseases [5]. The change from NAFLD to MAFLD improves the ability to identify individuals with metabolically complicated fatty liver and an increased risk of cardiovascular disease as well as significant hepatic fibrosis [6,7].

The increasing burden of MAFLD is primarily fueled by excess calorie intake and physical inactivity [5]. Moreover, according to the developmental origins hypothesis, early-life malnutrition may also increase one’s predisposition to metabolic diseases in later life [8]. Results from studies of the Dutch famine and the Ukraine famine have suggested that in utero exposure to starvation contributes to the development of type 2 diabetes in adulthood [9,10]. The Chinese Great Famine, which lasted from the late 1950s to the early 1960s, is regarded as one of the largest catastrophes in human history [11]. Previous epidemiological studies have shown an association between early-life exposure to the Chinese famine and the risk of NAFLD [12,13]. Famine exposure has also been associated with an increased risk of type 2 diabetes [14], obesity [15], and metabolic syndrome [16], all of which are closely intertwined with MAFLD. Even so, the evidence to date directly linking early famine exposure to MAFLD in adults is lacking and warrants further investigation.

In the present study, we used data from a cohort study of Chinese adults in Shanghai to examine the association between early-life famine exposure and the risk of MAFLD in adult life.

## 2. Methods

### 2.1. Study Population

The population-based cohort was established from June 2016 to December 2017 when 37,670 Chinese adults were enrolled from Songjiang District, Shanghai. A multistage, stratified, clustered sampling method was used to recruit participants from four communities (Zhongshan, Xinqiao, Sheshan, and Maogang) based on economic level and population size. One-third of the committees or villages were randomly selected from each community. In each committee or village, residents aged 20–74 years were invited into the cohort. The study design and sampling methods have been described in detail elsewhere [17,18].

**Figure 1:**
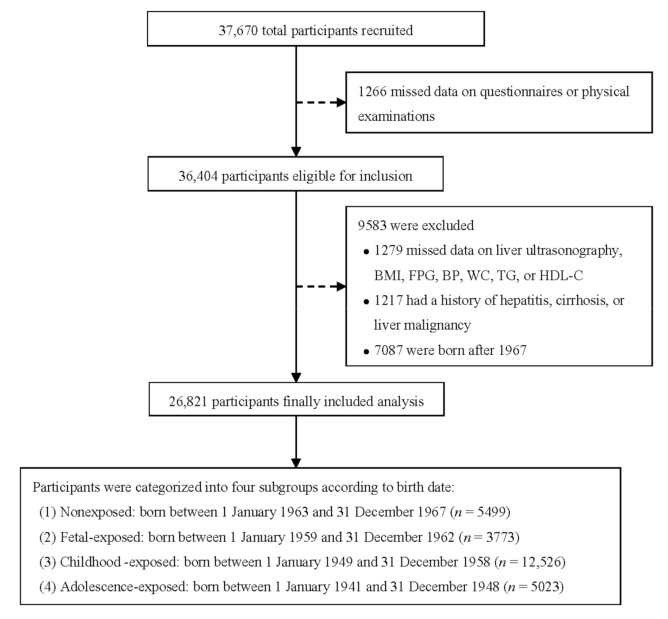
Flow chart of study participants. BMI, body mass index; BP, blood pressure; FPG, fasting plasma glucose; HDL-C, high-density lipoprotein cholesterol; TG, triglycerides; WC, waist circumference.

A total of 36,404 participants were recruited into the cohort with valid baseline data, comprising a questionnaire, physical measurements, and written informed consent. In the present study, we excluded participants who were born after 1967 (*n* = 7087), reported a history of hepatitis (*n* = 937), cirrhosis (*n* = 275), and liver malignancy (*n* = 5), had missing data on liver ultrasonography (*n* = 358) and physical measurements (*n* = 593), blood glucose, and lipids (*n* = 328). We finally included 26,821 participants in the analysis (Figure 1). All participants provided written informed consent and this study was approved by the Ethical Review Committee of the School of Public Health, Fudan University (IRB approval number 2016-04-0586).

### 2.2. Anthropometric and Biochemical Measurements

The survey used a standardized questionnaire that collected information about demographic characteristics (sex, age, education level, marital status), lifestyle factors (smoking, drinking, physical activity), and family history of chronic diseases (i.e., diabetes, hypertension). Height and weight were measured while participants were in light clothing and without shoes. Waist circumference (WC) was determined at the mid-point between the iliac crest and the last rib. Height and WC were precise to 0.1 cm and weight was precise to 0.1 kg. Blood pressure was measured three times using a digital sphygmomanometer on the right arm in a seated position after a 5 min rest. The mean value of three measurements was taken.

Venous blood samples were drawn in the morning after an overnight fast. The blood samples were shipped in dry ice within less than 6 h to the Shanghai Di’an Diagnostics Co Ltd. Alanine aminotransferase (ALT) was tested using IFCC AMP (Cobas c702 automatic biochemical analyzer, Roche Diagnostics, Basel, Switzerland). Serum lipids were tested using enzyme colorimetry (COBASC501 automatic biochemical analyzer, Roche Diagnostics, Basel, Switzerland). Fasting plasma glucose (FPG) was tested using the Glycokinase method (P800 automatic biochemical analyzer, Roche Diagnostics, Basel, Switzerland). Glycated hemoglobin (HbA1c) was measured using high pressure liquid chromatography (TOSOH G8, automatic hemoglobin A1c analyzer, Tosoh Bioscience, Tokyo, Japan).

### 2.3. Definition of Variables

Body mass index (BMI) was calculated as the weight in kilograms divided by height in meters squared (kg/m^2^). We determined obesity as BMI ≥ 28, overweight as BMI 24.0–27.9, normal as BMI 18.5–23.9, and underweight <18.5, according to Chinese criteria [19]. Hypertension was determined as systolic blood pressure (SBP) ≥ 140 mmHg, and/or diastolic blood pressure (DBP) ≥ 90 mmHg, and/or a self-reported previous diagnosis of hypertension. Type 2 diabetes was defined as FPG ≥ 7.0 mmol/L, and/or HbA1c ≥ 6.5%, and/or a self-reported previous diagnosis by health care professionals [20]. Dyslipidemia was defined as total cholesterol (TC) ≥ 6.22 mmol/L, triglycerides (TG) ≥ 2.26 mmol/L, low-density lipoprotein cholesterol (LDL-C) ≥ 4.14 mmol/L or high-density lipoprotein cholesterol (HDL-C) < 1.04 mmol/L, or a self-reported previous diagnosis of hyperlipidemia [21].

### 2.4. Definition of Famine Exposure

The Chinese Great Famine mainly lasted from 1959 to 1962. We categorized participants into four famine exposure subgroups according to the date of birth: nonexposed (born between 1 January 1963 to 31 December 1967), fetal-exposed (born between 1 January 1959 to 31 December 1962), childhood-exposed (born between 1 January 1949 to 31 December 1958), and adolescence-exposed (born between 1 January 1941 to 31 December 1948), as described in previous studies [13,14].

### 2.5. Ascertainment of Metabolic Associated Fatty Liver Disease

MAFLD was defined as liver steatosis detected by ultrasound in combination with one of the following three criteria: overweight/obesity, presence of type 2 diabetes, or evidence of metabolic dysregulation. In our study, metabolic dysregulation among thin/normal weight individuals with liver steatosis and who did not suffer from type 2 diabetes was determined by the presence of at least two of the following metabolic risk abnormalities [2]:

(1) Waist circumference ≥ 90 cm in men and 80 cm in women
(2) Blood pressure ≥ 130/85 mmHg or specific drug treatment
(3) TG ≥ 1.70 mmol/L or specific drug treatment
(4) HDL-C < 1.0 mmol/L for men and <1.3 mmol/L for women, or specific drug treatment
(5) Prediabetes (FPG levels of 5.6 to 6.9 mmol/L, and/or HbA1c levels of 5.7 to 6.4%).

### 2.6. Statistical Analysis

A one-way analysis of variance (ANOVA) or Student’s *t*-test and χ^2^ test were used to evaluate the differences in continuous and categorical variables across groups by famine exposure or MAFLD status. We used multivariable logistic regression models to estimate the association between early-life famine exposure and risk of MAFLD after adjusting for age (years), sex, education level (less than primary school, primary school, middle school, high school and above), marital status (married, widowed, divorced or separated, or never married), smoking (never, former, or current), drinking (never, former, or current), physical activity (none, mild, or moderate to vigorous), family history of diabetes (yes or no). To assess the potential sex-specific effects of famine exposure on MAFLD, analyses were also conducted separately in men and women. To reduce the bias related to age differences between famine exposure subgroups, an “age-balanced” method was adopted by combining post-famine and pre-famine births as the control group. Furthermore, considering the exact start and end dates of the Chinese Great Famine are unclear, we repeated the analyses by excluding participants born in 1959 and 1962 to minimize potential exposure misclassification. All statistical analyses were performed using IBM SPSS Statistics, version 22 (IBM Corp). All analyses were two-sided, *p* < 0.05 was declared as a significant difference.

## 3. Results

### 3.1. Baseline Characteristics of Participants

Of the 26,821 participants, 10,994 (41.0%) were men, and the mean ± SD age was 60.8 ± 6.8 years. The characteristics of participants according to famine exposure in early life are shown in Table 1. The proportions of the study population that have been exposed to the Chinese Great Famine during fetal time, childhood, and adolescence were 14.1%, 46.7%, and 18.7%, respectively. Compared with nonexposed and fetal-exposed participants, childhood-exposed and adolescent-exposed participants were more likely to be less educated and current drinkers, and had higher BMI, but had a lower proportion of family history of diabetes and hypertension. In general, famine-exposed participants had a higher prevalence of diabetes and hypertension.

The prevalence of MAFLD in the whole cohort is 39.2%. In comparison with participants without MAFLD, participants with MAFLD were a little younger and less likely to be men and current smokers and had a higher proportion of family history of diabetes and hypertension (Table 2). Undoubtedly, participants with MAFLD had poorer metabolic measures, including higher BMI, BP, FBG, ALT, TC, TG, and lower HDL-C than their non-MAFLD counterparts.

### 3.2. Association of Early-Life Famine Exposure with MAFLD

**Figure 2:**
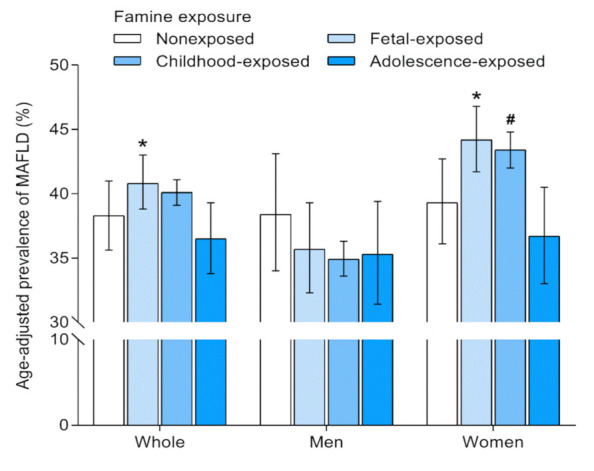
Age-adjusted prevalence of MAFLD according to famine exposure in early life. Prevalence rate was derived from logistic regression models. Error bars indicate 95% confidence intervals. MAFLD, metabolic associated fatty liver disease. * Compared with the unexposed, *p*-value < 0.05. ^#^ Compared with the unexposed, 0.05 < *p*-value < 0.1.

The age-adjusted prevalence of MAFLD according to famine exposure in early life is shown in Figure 2. In the whole cohort, the age-adjusted prevalence of MAFLD was 38.3% in nonexposed individuals, 40.8% in fetal-exposed individuals, 40.1% in childhood-exposed individuals, and 36.5% in adolescence-exposed individuals. When stratified by sex, the prevalence of MAFLD in fetal-exposed (44.2%) and childhood-exposed groups (43.4%) was higher in women; however, a lower prevalence of MAFLD in fetal-exposed and childhood-exposed groups than nonexposed group was observed in men.

In the logistic regression analyses adjusting for age, fetal exposure to famine was associated with an increased risk of adulthood MAFLD (Table 3). Compared with nonexposed participants, the age-adjusted ORs (95% CIs) of MAFLD were 1.11 (1.01–1.22), 1.08 (0.94–1.23), and 0.93 (0.75–1.15) for fetal-exposed, childhood-exposed, and adolescence-exposed participants, respectively. Further adjustments for sex, education level, marital status, smoking, drinking status, physical activity, and family history of diabetes had little effect on the results. The multivariable-adjusted OR (95% CI) of MAFLD comparing fetal-exposed versus nonexposed participants was 1.10 (1.00–1.21). Intriguingly, there was a divergent relationship of famine exposure with MAFLD between sexes (*p* for interaction < 0.001). A significant association between fetal famine exposure and MAFLD was observed in women (OR = 1.22, 95% CI 1.08–1.37), but not in men (OR = 0.88, 95% CI 0.75–1.03). When compared with pre-famine and post-famine births combined, women with fetal exposure to famine still had an increased risk of adulthood MAFLD (OR = 1.15, 95% CI 1.05–1.26). In the sensitivity analysis, the results did not change appreciably after excluding individuals born in 1959 and 1962 (OR = 1.28, 95% CI 1.10–1.50).

## 4. Discussion

In this cohort study of Chinese adults, fetal exposure to famine was associated with an increased risk of adulthood MAFLD. This association was predominantly present in women, but not in men. Our findings confirm and extend the existing evidence about the relationship between early-life famine exposure and later metabolic disorders such as NAFLD [12,13], metabolic syndrome [16], type 2 diabetes [14], and obesity [15].

Previous studies have indicated the association between exposure to famine in early life and the risk of NAFLD [12,13]. Results from a study of 10,935 Chinese adults reported that exposure to famine during fetal time and infancy was associated with an increased risk of fatty liver disease in adulthood [22]. A population-based study conducted in East China showed a sex-specific association between early-life famine exposure and NAFLD; women with exposure during the fetal and childhood period exhibited a higher risk of moderate to severe NAFLD in adult life [12]. Recently, Qi et al. explored the joint effects of famine exposure and adulthood obesity on NAFLD in a community-dwelling Chinese population and demonstrated the significant interaction between famine exposure and adulthood obesity on the development of NAFLD in women [13]. In the present study, we investigated the association of early-life famine exposure with MAFLD, a consensus-driven proposed nomenclature to more accurately describe the fatty liver disease associated with metabolic dysfunction such as type 2 diabetes, obesity, and dyslipidemia given the common coexistence of these abnormalities [2,5]. Our study for the first time found that fetal exposure to famine was associated with an increased risk of adulthood MAFLD, and such an association appeared to exist in women only. Our results suggest the fetal stage as a critical time period, which is susceptible to nutritional condition, for determining the long-term risk of MAFLD in adulthood.

The relation between early-life famine exposure and MAFLD in adulthood has some biological plausibility. According to the developmental origins hypothesis, adaptations in response to undernutrition in the fetus may cause long-term metabolic changes [8]. Accumulating evidence suggests that maternal undernutrition could induce the occurrence of fatty liver in rat offspring via activation of the key enzyme and up-regulation of gene expression related to lipid synthesis (i.e., SREBP-1c) [23,24]. Moreover, maternal undernutrition in animal models led to permanent changes in the mass and function of pancreatic β-cells and the tissues’ sensitivity to insulin [25]. Epigenetic modulation has been proposed as a possible mechanism linking early nutrition and long-term health outcomes [26]. The Dutch Famine Birth Cohort study showed that individuals with prenatal exposure to famine had differential DNA methylation of the imprinted IGF2 gene and other candidate genes involved in growth and metabolism [27,28]. Specific epigenetic influences of early-life undernutrition have been observed across numerous organs and pathways associated with metabolism [29].

In the current study, we found a sex-specific association whereby fetal exposure to famine was associated with MAFLD in women only. Consistently, there were similar sex differences in the association of early-famine exposure with type 2 diabetes, NAFLD, and metabolic syndrome [12,30,31]. The sex-dependent results might be related to the differential modulation of the risks of metabolic diseases such as type 2 diabetes and NAFLD by endogenous sex hormones in men and women. For example, low testosterone levels are associated with lower risks of type 2 diabetes and NAFLD in women but with higher risks in men [32,33]. In addition, estradiol was reported to be associated with type 2 diabetes in women but not in men [34,35]. Besides, prenatal exposure to famine was shown to trigger sex-specific changes in DNA methylation [36]. In addition, the effect of mortality selection may affect the relationship between early-life exposure and health outcomes between sexes. It has been shown that the better health status of male survivors most plausibly reflects higher male excess mortality than females during the famine in early life, which may have masked the true health impact of famine exposure on males later in life. Further studies are needed to elucidate the mechanisms underlying the sex differences on the effects of famine.

To the best of our knowledge, this study is the first to investigate the relationship between early-life famine exposure and the risk of MAFLD in adulthood. The strengths of our study include a relatively large sample size and comprehensive adjustment for potential confounding factors. The inclusion of participants from multiple communities using a multistage, stratified, clustered sampling method makes our sample representative. However, our study also has several limitations. First, the exact date of the start and end of the Chinese Great Famine is unclear, thus misclassification of famine exposure was inevitable. Nevertheless, this misclassification should be nondifferential and would attenuate our results. Additionally, our results were consistent even after excluding participants born in 1959 and 1962. Second, the fatty liver diagnosis was determined using ultrasound, which is incapable of detecting mild steatosis. Even so, ultrasound is the most common technique used for diagnosing fatty liver in population-based settings given its wide availability and affordability [37]. Third, although an age-balanced analysis provided more convincing results, it is still hard to eliminate the aging effect. Finally, information on maternal health and childhood growth as well as laboratory markers such as apolipoprotein, leptin, and adiponectin are lacking, thus the residual or unmeasured confounding cannot be ruled out.

## 5. Conclusions

Our study showed that fetal exposure to famine was associated with an increased risk of MAFLD in women. This finding may partly suggest sex-specific consequences of early-life undernutrition in the development of MAFLD later in life. Our study provides evidence supporting active intervention measures for individuals who experienced undernutrition in early life to prevent or alleviate the risk of MALFD.

## Figures and Tables

**Table 1:** Characteristics of 26,821 participants according to famine exposure in early life.

	Famine Exposure			
	Nonexposed	Fetal-Exposed	Childhood-Exposed	Adolescence-Exposed
No. of participants (%)	5499 (20.5)	3773 (14.1)	12,526 (46.7)	5023 (18.7)
Age at baseline (years)	51.5 ± 1.5	55.3 ± 1.3	62. 6 ± 2.9	70.5 ± 1.8
Male (%)	1904 (34.6)	1344 (35.6)	5395 (43.1)	2351 (46.8)
High school and above (%)	516 (9.4)	931 (24.7)	1014 (8.1)	194 (3.9)
Married (%)	5300 (96.4)	3629 (96.2)	11,695 (93.4)	4335 (86.3)
Current smoker (%)	1101 (20.0)	776 (20.6)	2647 (21.1)	910 (18.1)
Current drinker (%)	615 (11.2)	413 (11.0)	1840 (14.7)	716 (14.3)
Moderate and vigorous physical activity (%)	1203 (21.9)	859 (22.8)	3138 (25.1)	1121 (22.3)
Family history of diabetes (%)	786 (14.3)	563 (14.9)	1212 (9.7)	365 (7.3)
Family history of hypertension (%)	2684 (48.8)	1831 (48.5)	4792 (38.3)	1455 (29.0)
BMI (kg/m^2^)	24.5 ± 3.2	24.5 ± 3.2	24.6 ± 3.20	24.9 ± 3.5
SBP (mmHg)	132.2 ± 17.8	133.9 ± 18.5	137.5 ± 19.0	140.8 ± 19.6
DBP (mmHg)	81.3 ± 10.7	81.1 ± 10.2	81.0 ± 9.9	79.7 ± 10.0
FBG (mmol/L)	4.99 ± 1.19	5.18 ± 1.52	5.25 ± 1.50	5.13 ± 1.59
ALT (IU/L)	18 (13–25)	18 (14–25)	17 (14–23)	17 (13–22)
TC (mmol/L)	5.00 ± 0.92	5.08 ± 0.97	5.08 ± 0.97	4.97 ± 0.96
TG (mmol/L)	1.43 (1.03–2.05)	1.45 (1.06–2.07)	1.40 (1.03–1.96)	1.30 (0.96–1.81)
HDL-C (mmol/L)	1.39 ± 0.34	1.39 ± 0.34	1.41 ± 0.36	1.45 ± 0.38
LDL-C (mmol/L)	2.80 ± 0.83	2.87 ± 0.86	2.85 ± 0.85	2.83 ± 0.86
Diabetes (%)	611 (11.1)	633 (16.8)	2312 (18.5)	963 (19.2)
Hypertension (%)	2528 (46.0)	1955 (51.8)	7909 (63.1)	3666 (73.0)
Dyslipidemia (%)	1978 (20.4)	1446 (14.9)	4573 (47.1)	1711 (17.6)
MAFLD (%)	2243 (40.8)	1597 (42.3)	4960 (39.6)	1708 (34.0)

Data are presented as mean ± SD or median (IQR) or number (percentage). ALT, alanine aminotransferase; BMI, body mass index; DBP, diastolic blood pressure; HDL-C, high-density lipoprotein cholesterol; LDL-C, low-density lipoprotein cholesterol; MAFLD, metabolic associated fatty liver disease; SBP, systolic blood pressure; TC, total cholesterol; TG, triglycerides. All *p* values are <0.05 for comparison across groups. FBG, fasting plasma glucose.

**Table 2:** Characteristics of 26,821 participants according to MAFLD status.

	MAFLD	Non-MAFLD
No. of participants (%)	10,508 (39.2)	16,313 (60.8)
Age at baseline (years)	60.4 ± 6.7	61.0 ± 6.9
Male (%)	3945 (37.5)	7049 (43.2)
High school and above (%)	1060 (10.1)	1595 (9.8)
Married (%)	9850 (93.7)	15,109 (92.6)
Current smoker (%)	1891 (18.0)	3543 (21.7)
Current drinker (%)	1369 (13.0)	2215 (13.6)
Moderate and vigorous physical activity (%)	2610 (24.8)	3711 (22.8)
Family history of diabetes (%)	1355 (12.9)	1571 (9.6)
Family history of hypertension (%)	4622 (44.0)	6140 (37.6)
BMI (kg/m^2^)	26.7 ± 2.9	23.3 ± 2.8
SBP (mmHg)	140.3 ± 18.5	134.1 ± 18.9
DBP (mmHg)	82.7 ± 9.9	79.6 ± 10.1
FBG (mmol/L)	5.43 ± 1.70	5.00 ± 1.27
ALT (IU/L)	20 (16–28)	16 (13–21)
TC (mmol/L)	5.10 ± 0.99	4.96 ± 0.91
TG (mmol/L)	1.73 (1.27–2.42)	1.22 (0.92–1.64)
HDL-C (mmol/L)	1.30 ± 0.31	1.48 ± 0.36
LDL-C (mmol/L)	2.86 ± 0.89	2.83 ± 0.82
Diabetes (%)	2732 (26.0)	1787 (11.0)
Hypertension (%)	7503 (71.4)	8555 (52.4)
Dyslipidemia (%)	5360 (51.0)	4348 (26.7)

Data are presented as mean ± SD or median (IQR) or number (percentage). ALT, alanine aminotransferase; BMI, body mass index; DBP, diastolic blood pressure; HDL-C, high-density lipoprotein cholesterol; LDL-C, low-density lipoprotein cholesterol; MAFLD, metabolic associated fatty liver disease; SBP, systolic blood pressure; TC, total cholesterol; TG, triglycerides. All *p* values are <0.05 for comparison across groups, with the exception of education level and drinking status.

**Table 3:** ORs (95% CIs) for MAFLD according to famine exposure in early life.

	Famine Exposure			
	Nonexposed	Fetal-Exposed	Childhood-Exposed	Adolescence-Exposed
Whole cohort				
Case/total (*n*)	2243/5499	1597/3773	4960/12526	1708/5023
Age-adjusted	1.00 (ref)	1.11 (1.01–1.22)	1.08 (0.94–1.23)	0.93 (0.75–1.15)
Multivariable-adjusted ^a^ *p*	1.00 (ref)	1.10 (1.00–1.21) 0.049	1.07 (0.93–1.22) 0.362	0.91 (0.73–1.14) 0.409
Men				
Case/total (*n*)	871/1904	539/1344	1843/5395	692/2351
Age-adjusted	1.00 (ref)	0.89 (0.76–1.04)	0.86 (0.69–1.07)	0.87 (0.62–1.23)
Multivariable-adjusted *p*	1.00 (ref)	0.88 (0.75–1.03) 0.122	0.85 (0.68–1.06) 0.145	0.86 (0.61–1.21) 0.386
Women				
Case/total (*n*)	1372/3595	1058/2429	3117/7131	1016/2672
Age-adjusted	1.00 (ref)	1.22 (1.09–1.38)	1.18 (0.99–1.41)	0.89 (0.67–1.19)
Multivariable-adjusted *p*	1.00 (ref)	1.22 (1.08–1.37) 0.001	1.16 (0.98–1.38) 0.097	0.88 (0.66–1.17) 0.387

^a^ Adjusted for age, sex (for the whole cohort), education level (less than primary school, primary school, middle school, high school and above), marital status (married, widowed, divorced or separated, or never married), smoking status (never, former, or current), drinking status (never, former, or current), physical activity (none, mild, or moderate to vigorous), family history of diabetes (yes or no).

## Data Availability

The dataset used and analyzed during the current study is available from the corresponding author upon reasonable request.
